# Symptoms of mental health problems among Italian adolescents in 2017–2018 school year: a multicenter cross-sectional study

**DOI:** 10.1186/s12199-021-00988-4

**Published:** 2021-06-21

**Authors:** Francesco Donato, Maria Triassi, Ilaria Loperto, Alessia Maccaro, Sara Mentasti, Federica Crivillaro, Antonella Elvetico, Elia Croce, Elena Raffetti

**Affiliations:** 1 Unit of Hygiene, Epidemiology and Public Health, University of Brescia, v.le Europa, 11, 25121 Brescia, Italy; 2 Department of Public Health, University of Naples “Federico II”, Via S. Pansini, 5, 80131 Naples, Italy; 3 Institute of Advanced Study, University of Warwick, Coventry, CV4 7AL UK; 4 Fondazione Poliambulanza Istituto Ospedaliero Multispecialistico, via Bissolati, 57, 25124 Brescia, Italy; 5 Epidemiology and Public Health Intervention Research Group (EPHIR), Department of Global Public Health, Karolinska Institutet, Solnavägen 1 E, 11365 Stockholm, Sweden

**Keywords:** Mental health, Risk factors, Behaviors, Social context

## Abstract

**Background:**

Identifying individual and contextual factors that influence adolescent well-being is a research priority. This study aimed to assess the prevalence of symptoms of mental health problems and some related factors in Italian adolescents in 2017–2018.

**Methods:**

The present study was a cross-sectional survey among 3002 students aged 15–16 years who resided in two Italian provinces, in North and South Italy. Symptoms of mental health problems were assessed using the SDQ and CES-DC, and students’ risk-taking behaviors and school climate perception were assessed. All information was collected anonymously. Logistic regression models were used to assess the associations of tobacco and alcohol use, screen time, bullying, and school climate with symptoms of mental health problems.

**Results:**

One student out of five reported symptoms of mental health problems, with a more than double proportion among girls than boys (28.7% vs 10.4% with depressive symptoms, respectively). Thirty percent and 40% of students smoked tobacco or drank alcoholic beverages at least once in the past month, and more than 40% reported being victims or authors of bullying in the past 6 months. Smoking behavior, alcohol consumption, screen time, bullying, and negative school climate had 1.2- to 3.3-fold increased odds of symptoms of mental health problems without substantial differences between sexes and geographical areas.

**Conclusions:**

Tobacco and alcohol use, screen time, bullying, and school climate were independently associated with symptoms of mental health problems in a large sample of 15–16-year-old Italian adolescents without substantial gender and geographical differences.

**Supplementary Information:**

The online version contains supplementary material available at 10.1186/s12199-021-00988-4.

## Background

The prevalence of depression and anxiety disorders is one of the main causes of disability-adjusted life years among adolescents and young adults [1]. In 2018, depressive disorders affected 3.6% of adolescents (15–19 years of age) in Italy, with a preponderance of females (4.7% females and 2.5% males) [2]. Adolescence is a crucial period for brain development, the onset of risk-taking behaviors, and mental health problems [3]. Although prevention of depression and other psychiatric disorders is a key point in preventive medicine strategies, the underlying causal pathway that leads to the onset of mental health problems among adolescents is complex and involves the interplay of several factors such as family history of depression, exposure to psychosocial stress, developmental factors, sex hormones, and psychosocial adversities [3].

Substance use, particularly tobacco smoking, use of alcohol, cannabis, and other illicit drug, is considered an important determinant of subsequent development of mental health problems among adolescents [4, 5]. Social context along with school context may influence the onset of psychiatric disorders due to negative interactions with peers [3] or school failure [6, 7]. Victimization from bullying is another risk factor for mental health problems and, conversely, mental health problems may predispose adolescents to bullying victimization [8, 9]. The use of technologies during leisure time, including television, social media, videogames, and the internet has rapidly increased among adolescents in the 2000s. Screen time has been associated with mental health problems in adolescence in some studies, although overall findings are inconsistent, with substantial differences according to types of screen time and non-linear time-varying associations [10, 11].

Although prevention of adolescents’ mental health problems is of the utmost importance, present evidence of the role of modifiable risk factors is uncertain. Differences in time period and societal context may explain, at least partly, the discrepancies on factors influencing adolescents’ mental health between studies, when also considering the rapid changes in adolescents’ behaviors, particularly as regards the use of technology devices and social relationships, in the 2000s.

The present study aimed to evaluate the prevalence of symptoms of mental health problems and related risk factors in Italian high school students in 2017–2018, few years before the beginning of the COVID-19 pandemic.

## Materials and methods

### Study design

The Be Teen project is an epidemiological study set up to investigate the determinants of symptoms of mental health problems among high school students in Brescia and Naples provinces; two different areas of Italy in term of geographical location, social interaction, and industrialization.

Naples and Brescia provinces are densely populated metropolitan areas located in the south and north part of Italy, respectively. Naples compared to Brescia province is a less industrialized area and has a higher prevalence of tobacco smoking (26.5% vs. 22.6%), obesity (13.8% vs. 7.5%), depressive symptoms (7.6% vs 5.8%), and unemployment (44.2% vs 29.5%) but lower alcohol consumption (44.4% vs. 60.8%) in the adult population, according to a recent national survey (2016–2019) [12].

This study has applied the same methodology as the Swedish Kupol study [13] and includes both a cross-sectional and a prospective cohort study. The present paper reports the results of the cross-sectional survey, whereas those of the cohort study will be shown at the end of the follow-up, in 2022. The study was conducted in accordance with the guidelines of the Declaration of Helsinki and the principles of Good Clinical Practice. It was approved by the Ethics Committee of the Brescia province (Dnr: 2761-04-10-2017) and ethics Committee “Carlo Romano”, University of Naples “Federico II” (Dnr: 272/17).

**Fig. 1:**
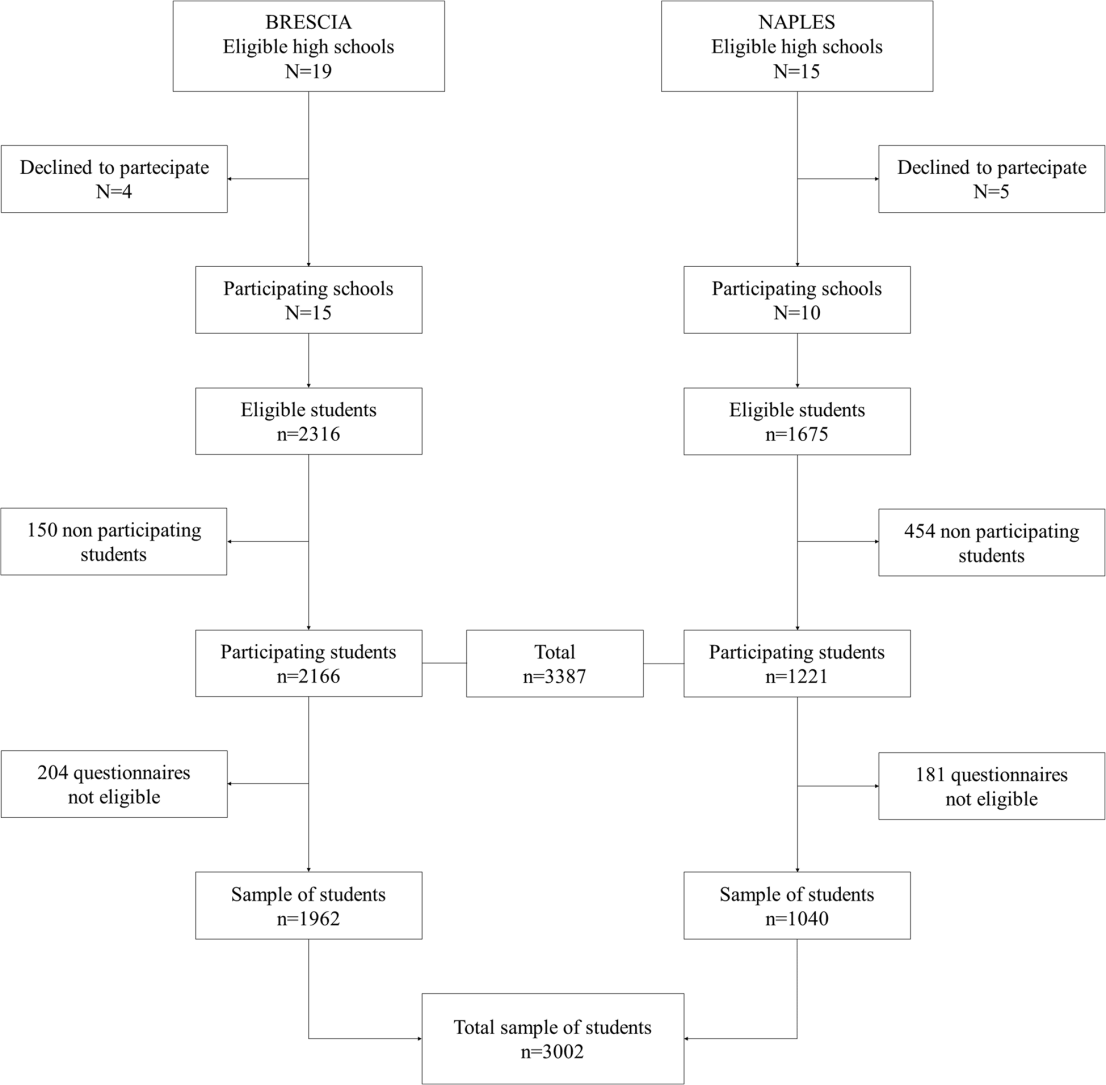
Flow chart for inclusion of study participants

The schools were recruited in 2017. Inclusion criteria for the institutes were to be located in Brescia and Naples provinces, to include at least 20 students per school class and at least 5 classes for 10th grade. For each institute, 10th-grade students, resident in the province, aged 15–16 years and able to understand every item of the questionnaire were eligible. The survey was carried out between November 2017 and November 2018. The flowchart of the selection of schools and students and their participation is shown in Fig. 1. Nineteen of the 39 public and private schools in Brescia Province were considered eligible and contacted, and 15 of them (79%) adhered to the project. Two thousand three hundred sixteen students attending the selected schools were invited to participate: 2166 of them accepted (93%) and 1962 provided complete data. In Naples city and province, a random sample of eligible school was invited. Among them, 10 adhered to the project (67%), and 1221 out of 1675 students invited (73%) accepted to enter the survey: 1040 of them provided complete data. Overall, a total of 3002 students were included in the present study: 1962 from Brescia and 1040 from Naples Province.

### Questionnaire

The school personnel could choose between computer- and paper-based anonymous questionnaires. The questionnaire consisted of 27 items investigating age and gender, parental education, mental health, school climate, tobacco smoking and alcohol consumption, bullying, and use of technology devices during leisure time (television, social networks, web navigation, videogames).

### Symptoms of mental health problems

Mental health was evaluated through two scales for psychological wellness: CES-DC (Center for Epidemiological Studies-Depression Scale for Children) and SDQ (Strengths and Difficulties Questionnaire). The CES-DC is a 20-item self-administered questionnaire that investigates the prevalence of symptoms linked to depression during the past 7 days [14]. The score ranges from 0 to 60 and a cut-off score of 30 or higher indicates the presence of depressive symptoms.

The SDQ is a 25-item self-report questionnaire evaluating different psychological symptoms and behaviors in the past 6 months [15]. It consists of 5 subscales, each one of 5 items: emotional symptoms, conduct problems, hyperactivity-inattention, peer problems, and prosocial behavior. The prosocial behavior subscale score reflects psychological strengths, whereas the total difficulties score is the sum of the other subscales’ scores. SDQ screening for mental health problems uses the total difficulties scale. Dichotomizing the SDQ score, we considered 16 as the cut-off for detecting symptoms of mental health problems. In addition, we evaluated two SDQ sub-scores: the internalizing and the externalizing scores, the former concerning peer relationship problems and emotional symptoms, and the latter behavior problems and hyperactivity/inattention [16]. A score above 9 and above 11 was considered as the threshold for internalizing and externalizing scales, respectively.

Both CES-DC and SDQ have been internationally validated as screening questionnaires for symptoms of mental health disorders. In this sample, the Cronbach alpha was 90.7% and 78.4% for CES-DC and SDQ, respectively.

### Risk factors for mental health

Students were considered current tobacco smokers if they had smoked at least one cigarette in the past 30 days and perceived tobacco dependence if they have ever perceived tobacco dependence. Alcohol consumption and perceived drunkenness were both investigated in the past month: the former as drinking at least one alcoholic beverage, and the latter as the perception of drunkenness. The legal ages for tobacco use and alcohol consumption are 18 and 16 years, respectively, in Italy.

The use of technology devices during leisure time was investigated as hours spent during the weekdays and weekends in watching television, navigating the web, playing videogames, and using social networks. For each type of screen time, mean daily time was considered. Bullying was evaluated through questions about general, psychological, and physical bullying in the past 6 months, as a victim or author. The school climate was investigated through the PESOC questionnaire (Pedagogical and Social Climate of a school) [17]. It contains 53 positive items on the school context and assesses students’ perceptions of 8 dimensions regarding their school: expectations, perception of teacher norms, teachers’ support, teaching activities, student participation, school environment, school and home, and school management [17]. The score ranged from 1 to 4 for each domain, a mean score of PESOC item sum of 2.5 was considered as the cut-off for negative school climate.

### Other covariates

Parental education was defined as the highest educational degree achieved by any parent (elementary school, high school, and university) and included as a possible confounder.

### Statistical analysis

A descriptive analysis of the characteristics of the sample was performed. Separate analyses were conducted for gender and center. Categorical variables were reported as proportions and continuous variables as means and standard deviations. The sample characteristics were compared by gender and center using common statistical tests: the Wilcoxon rank test for continuous variables, due to non-normal distribution, and the chi-square test for proportions. For the ordinal screen time variables, a test for linear trend was performed.

The associations between risk factors and mental health problems were evaluated using logistic regression models with symptoms of mental health problems, according to CES-DC and SDQ scales and subscales, as the dichotomous dependent variable. Each risk factor was examined using three models: unadjusted model, adjusted model A, and adjusted model B. Adjusted model A is a logistic regression model that accounts for possible confounders, including sex, parental education, tobacco smoking, social networks, and bullying victimization. Adjusted multilevel model B includes model A confounders and clusters on institutes and centers. The odds ratios (ORs) were computed as measures of association with their 95% confidence intervals (95% CIs). For all the analyses, two-tailed statistical tests were performed at the level of 0.05. All the statistical analyses were performed using the Stata software version 14.0 (StataCorp, College Station, TX, USA).

## Results

A total of 3002 adolescents were included, 55.9% females, with a mean age of 15.2 years old. The characteristics of the students by gender are set out in Table 1. 46.5% of the students had at least one parent with a high school degree and 38.6% a university degree. 30.4% of the students reported current smoking, and 13% perception of having been tobacco dependent. Alcohol consumption was more prevalent than tobacco smoking, as it was reported by 40.4% of students, especially among males (46.5%). Regarding screen time, students used social networks more often than other technology tools. The daily time for social media equal or higher than 4 h was referred by 45.1% of the students, with an overall higher exposure in females than males. Bullying was reported by about 40% of the students (41.4% victims and 39.8% authors), with a higher proportion of victims among females than males (43.7% vs 38.4%), whereas more males than females referred to have been authors (48.4% vs 33.1%). Finally, one out of three students had a negative perception of their school climate. Students with CES-DC and SDQ with symptoms of mental health problems were 20.8% and 17.8%, respectively, with a higher proportion of females than males (28.7% vs 10.4% for CES-DC and 22.3% vs 12.1% for SDQ, respectively). The two scores were highly related with 0.739 linear correlation coefficient between them.

**Table 1:** Characteristics of students according to gender

	Females	Males	All	
	n (%)	n (%)	n (%)	***p*** value^a^
**Total subjects**	1679 (55.9)	1323 (44.1)	3002	
**Geographical area**				
Brescia	1056 (62.9)	906 (68.5)	1962 (65.4)	
Naples	623 (37.1)	417 (31.5)	1040 (34.6)	0.002
**Mean age (SD), years**	15.2 (0.4)	15.2 (0.4)	15.2 (0.4)	
**Higher parental education, years**				
≤ 8	249 (15.3)	180 (14.4)	429 (14.9)	
9–13	793 (48.8)	546 (43.5)	1339 (46.5)	
≥ 14	582 (35.8)	528 (42.1)	1110 (38.6)	
**Substance use§**				
Tobacco smoking	529 (31.5)	383 (29.0)	912 (30.4)	0.142
Perceived tobacco dependence	239 (14.7)	140 (10.8)	379 (13.0)	0.002
Alcohol consumption	586 (35.6)	603 (46.5)	1189 (40.4)	< 0.001
Perceived drunkenness	98 (6.0)	107 (8.4)	205 (7.1)	0.015
**Screen time**				
TV				
0–1 h	835 (51.0)	786 (61.4)	1621 (55.6)	
2–3 h	448 (27.4)	302 (23.6)	750 (25.7)	
≥ 4 h	353 (21.6)	192 (15.0)	545 (18.7)	<0.001
Mean (SD), h	2.09 (1.52)	1.73 (1.43)	1.93 (1.49)	
Social networks				
0–1 h	324 (19.7)	570 (44.0)	894 (30.4)	
2–3 h	393 (23.9)	328 (25.4)	721 (24.5)	
4–5 h	419 (25.4)	235 (18.2)	654 (22.3)	
≥ 6 h	511 (31.0)	160 (12.4)	671 (22.8)	< 0.001
Mean (SD), h	3.8 (2.1)	2.5 (1.9)	3.27 (2.1)	
Web navigation				
0–1 h	1211 (74.2)	1050 (82.4)	2261 (77.8)	
2–3 h	258 (15.8)	149 (11.7)	407 (14.0)	
≥ 4 h	163 (10.0)	75 (5.9)	238 (8.2)	< 0.001
Mean (SD), h	1.42 (1.3)	1.12 (1.1)	1.28 (1.2)	
Videogames				
0–1 h	1531 (93.7)	723 (56.2)	2254 (77.2)	
2–3 h	50 (3.1)	292 (22.7)	342 (11.7)	
≥ 4 h	53 (3.2)	271 (21.1)	324 (11.1)	< 0.001
Mean (SD), h	0.4 (1.1)	1.93 (1.8)	1.08 (1.6)	
**Screen time$**				
Mean (SD), h	1.5 (01.0)	1.6 (1.0)	1.55 (1.0)	
**Bullying**				
Bullying				
Victims	728 (43.7)	502 (38.4)	1230 (41.4)	0.004
Authors	551 (33.1)	628 (48.4)	1179 (39.8)	< 0.001
Physical				
Victims	78 (4.7)	120 (9.2)	198 (6.7)	< 0.001
Authors	99 (6.0)	240 (18.8)	339 (11.5)	< 0.001
Psychological				
Victims	637 (38.6)	378 (29.1)	1015 (34.4)	< 0.001
Authors	445 (26.8)	379 (29.6)	824 (28.1)	0.095
**School climate**				
Negative perception	567 (33.8)	415 (31.4)	982 (32.7)	0.172
**Mental health evaluation**				
CES-DC scale				
Depressive symptoms	432 (28.7)	119 (10.4)	551 (20.8)	< 0.001
Mean (SD)	22.50 (12.8)	14.86 (10.9)	19.20 (12.6)	< 0.001
**SDQ scale, overall**				
Mean (SD)	14.62 (6.1)	12.01 (6.0)	13.48 (6.2)	< 0.001
Symptoms of mental health problems	366 (22.3)	155 (12.1)	521 (17.8)	< 0.001
**SDQ scale, internalizing subscale**				
Internalizing symptoms	580 (35.1)	207 (16.1)	787 (26.7)	< 0.001
Mean (SD)	7.23 (3.8)	4.96 (3.6)	6.24 (3.9)	< 0.001
**SDQ scale, externalizing subscale**				
Externalizing symtoms	326 (19.7)	211 (16.3)	537 (18.2)	0.020
Mean (SD)	7.4 (3.5)	7.1 (3.5)	7.3 (3.5)	0.005

^a^ p value refers to χ2 test or test for linear trend of the log-odds for categorical variables and Wilcoxon rank test for continuous variables

^$^Total time spent in TV, social networks, web navigation, or videogames

^§^Tobacco or alcohol use or perception: at least once in previous month

Supplementary Table 1 shows the characteristics of students separated between centers. The prevalence of symptoms of mental health problems was higher among Naples than Brescia students (32.4% vs 26.5% for CES-DC). More students from Brescia than Naples reported substance use: for tobacco smoking only among males (females 33.1% vs 28.9%; males 31.2% vs 24.2%) and for alcohol consumption in both genders (females 39.1% vs 29.7%; males 48.4% vs 42.0%). More girls from Brescia than from Naples reported active bullying (36.2 and 27.7%, respectively). Using social network for more than 6 h per day was more frequent among Naples than Brescia students in both females (46.6% vs 21.9%) and males (23.8% vs 7.3%).

The associations between depressive symptoms (CES-DC score) and risk factors are shown in Table 2. Tobacco and alcohol use, screen time, physical and psychological bullying, and negative school climate perception were associated with depressive symptoms with ORs ranging from 1.2 to 3.3. Among the technology devices, stronger associations with symptoms of mental health problems were found for TV and social network use than web navigation and videogame. The OR estimates did not change substantially from unadjusted to adjusted models. Similar associations were found in both males and females, when considered separately (Supplementary Tables 2 and 3).

**Table 2:** Number and proportion of students with, and odds ratio for, depressive symptoms on CES-DC according to behavioral factors and school climate perception

Independent variables		n (%)	Unadjusted models	Adjusted models A	Adjusted models B
			OR (95% CI)	OR (95% CI)	OR (95% CI)
**Gender**					
Females		432 (28.7)	1	1	1
Males		119 (10.4)	0.3 (0.2,0.4)	0.3 (0.3,0.4)	0.3 (0.3,0.4)
**Higher parental education, years**					
≤ 8		80 (21.7)	1	1	1
9–13		238 (20.1)	0.9 (0.7,1.2)	1.0 (0.8,1.4)	1.0 (0.7,1.4)
≥ 14		207 (20.5)	0.9 (0.7,1.2)	1.3 (0.9,1.7)	1.3 (0.9,1.7)
**Substance use**					
Tobacco smoking	No	311 (17.0)	1	1	1
	Yes	240 (29.1)	2.0 (1.6,2.4)	1.8 (1.5,2.3)	1.9 (1.5,2.3)
Perceived tobacco dependence	No	399 (17.7)	1	1	1
	Yes	126 (36.2)	2.6 (2.1,3.4)	2.2 (1.7,2.9)	2.3 (1.7,3.0)
Alcohol consumption	No	299 (19.4)	1	1	1
	Yes	244 (22.7)	1.2 (1.0,1.5)	1.4 (1.1,1.7)	1.4 (1.1,1.7)
Perceived drunkenness	No	477 (20.0)	1	1	1
	Yes	56 (29.2)	1.6 (1.2,2.3)	1.9 (1.3,2.7)	1.9 (1.3,2.8)
**Screen time**					
TV					
0–3 h		382 (18.0)	1	1	1
≥ 4 h		157 (32.7)	2.2 (1.8,2.8)	1.9 (1.5,2.5)	1.9 (1.5,2.4)
Social networks					
0–3 h		207 (14.4)	1	1	1
4–5 h		145 (24.5)	1.9 (1.5,2.4)	1.5 (1.1,1.9)	1.4 (1.1,1.8)
≥ 6 h		188 (31.9)	2.8 (2.2,3.5)	1.9 (1.4,2.4)	1.8 (1.3,2.3)
Web navigation					
0–3 h		472 (19.8)	1	1	1
≥ 4 h		67 (32.4)	1.9 (1.4,2.6)	1.6 (1.1,2.2)	1.5 (1.1,2.1)
Videogames					
0–3 h		490 (21.1)	1	1	1
≥ 4 h		48 (17.0)	0.8 (0.5,1.1)	1.3 (0.9,1.9)	1.2 (0.8,1.8)
**Bullying**					
Bullying					
Victims	No	207 (13.5)	1	1	1
	Yes	340 (30.6)	2.8 (2.3,3.4)	2.7 (2.2,3.4)	2.7 (2.2,3.4)
Author	No	305 (19.3)	1	1	1
	Yes	240 (22.7)	1.2 (1.0,1.5)	1.2 (1.0,1.5)	1.2 (1.0,1.5)
Physical bullying					
Victims	No	476 (19.4)	1	1	1
	Yes	64 (37.0)	2.4 (1.8,3.4)	3.2 (2.2,4.7)	3.3 (2.3,4.7)
Author	No	471 (20.3)	1	1	1
	Yes	63 (21.4)	1.1 (0.8,1.4)	1.3 (1.0,1.9)	1.4 (1.0,1.9)
Psychological bullying					
Victims	No	271 (15.9)	1	1	1
	Yes	269 (29.0)	2.2 (1.8,2.6)	2.0 (1.6,2.4)	2.0 (1.6,2.5)
Author	No	365 (19.5)	1	1	1
	Yes	169 (22.5)	1.2 (1.0,1.5)	1.1 (0.9,1.3)	1.1 (0.9,1.4)
**School climate**					
Positive perception		293 (16.4)	1	1	1
Negative perception		258 (29.7)	2.2 (1.8,2.6)	1.9 (1.6,2.4)	2.0 (1.6,2.5)

Models A: adjusted for tobacco smoking, social networks use, bullying victimization, parental education, and sex Models B: multilevel regression models for institutes and centers adjusted as Models A

Accordingly, the same variables were associated with symptoms of mental health problems according to SDQ score (Table 3), with OR estimates ranging from 1.3 to 5.0. The strongest association was observed for being a victim of physical bullying (ORs from 4.5 to 5.0). Yet, no substantial differences were found adjusting for covariates. Similar results were observed when stratifying for gender, apart from a moderately stronger association between physical bullying and symptoms of mental health problems among females than males (OR 5.5 vs 3.4). (Supplementary Tables 4 and 5).

**Table 3:** Number and proportion of students with, and odds ratio for, symptoms of mental health problems on SDQ according to behavioral factors and school climate perception

Independent variables		n (%)	Unadjusted models	Adjusted models A	Adjusted models B
			OR (95% CI)	OR (95% CI)	OR (95% CI)
**Gender**					
Males		155 (12.1)	1	1	1
Females		366 (22.3)	0.5 (0.4,0.6)	0.6(0.4, 0,7)	0.6 (0.4,0.7)
**Higher parental education, years**					
≤ 8		94 (22.7)	1	1	1
9–13		198 (15.1)	0.6 (0.5,0.8)	0.6 (0.5, 0.8)	0.6 (0.5,0.9)
≥ 14		200 (18.3)	0.8 (0.6,1.0)	1.0 (0.7,1.3)	1.0 (0.7,1.4)
**Substance use**					
Tobacco smoking	No	301 (14.8)	1	1	1
	Yes	220 (24.6)	1.9 (1.5,2.3)	1.7 (1.3,2.1)	1.7 (1.4,2.1)
Perceived tobacco dependence	No	382 (15.4)	1	1	1
	Yes	120 (32.3)	2.6 (2.1,3.3)	2.4 (1.8,3.2)	2.4 (1.9,3.2)
Alcohol consumption	No	277 (16.2)	1	1	1
	Yes	232 (19.9)	1.3 (1.1,1.6)	1.3 (1.1,1.6)	1.4 (1.1,1.7)
Perceived drunkenness	No	451 (17.1)	1	1	1
	Yes	48 (23.8)	1.5 (1.1,2.1)	1.5 (1.0,2.2)	1.5 (1.0,2.2)
**Screen time**					
TV					
0–3 h		349 (15.0)	1	1	1
≥ 4 h		161 (30.2)	2.5 (2.0,3.0)	2.4 (1.9,3.1)	2.3 (1.8,3.0)
Social networks					
0–3 h		199 (12.6)	1	1	1
4–5 h		123 (19.2)	1.7 (1.3,2.1)	1.4 (1.1,1.8)	1.3 (1.0,1.8)
≥ 6 h		191 (29.1)	2.8 (2.3,3.6)	2.2 (1.7,2.9)	2.1 (1.6,2.7)
Web navigation					
0–3 h		436 (16.6)	1	1	1
≥ 4 h		70 (30.3)	2.2 (1.6,2.9)	1.9 (1.4,2.7)	1.8 (1.3,2.4)
Videogames					
0–3 h		439 (17.2)	1	1	1
≥ 4 h		66 (21.2)	1.3 (1.0,1.7)	1.8 (1.3,2.6)	1.7 (1.2,2.4)
**Bullying**					
Bullying					
Victims	No	186 (10.9)	1	1	1
	Yes	328 (27.2)	3.1 (2.5,3.7)	3.1 (2.5,3.9)	3.1 (2.5,3.9)
Author	No	236 (13.4)	1	1	1
	Yes	277 (24.0)	2.0 (1.7,2.5)	2.1 (1.7,2.6)	2.1 (1.7,2.6)
Physical bullying					
Victims	No	420 (15.6)	1	1	1
	Yes	88 (45.4)	4.5 (3.3,6.1)	5.0 (3.5,7.0)	5.0 (3.6,7.0)
Author	No	409 (16.0)	1	1	1
	Yes	96 (29.3)	2.2 (1.7,2.8)	2.7 (2.0,3.7)	2.7 (2.0,3.7)
Psychological bullying					
Victims	No	274 (14.4)	1	1	1
	Yes	234 (23.5)	1.8 (1.5,2.2)	1.9 (1.5,2.3)	1.9 (1.5,2.3)
Author	No	328 (15.9)	1	1	1
	Yes	177 (21.8)	1.5 (1.2,1.8)	1.4 (1.1,1.7)	1.4 (1.1,1.8)
**School climate**					
Positive perception		283 (14.4)	1	1	1
Negative perception		238(24.7)	1.9 (1.6,2.4)	1.7(1.4,2.1)	1.8(1.4,2.2)

Models A: adjusted for tobacco smoking, social networks use, bullying victimization, parental education, and sex Models B: multilevel regression models for institutes and centers adjusted as Models A

## Discussion

The main result of this study was the relatively high proportion of 15–16 years old adolescents with symptoms of mental health problems at screening questionnaires, higher in girls than in boys, particularly regarding internalizing symptoms. About one-third of the students claimed to have smoked tobacco and almost half of males had consumed alcohol in the past month. Several students spent a long time on social networks every day, and more than 40% of students said to have been victims or authors of bullying in the past 6 months. Finally, substance use, particularly tobacco smoking, screen time, and bullying were all associated independently with an increased odds of symptoms of mental health problems for both CES-DC and SDQ scales. Associations were in the same direction also stratifying by sex.

Differences in study design, population characteristics, instruments used for defining mental health problems, and study period may challenge comparisons. The findings of some recent studies which assessed depressive or emotional symptom prevalence in 15–16 years old adolescents are reported in Table 4. Findings similar to ours were reported in the Swedish Kupol study, which was carried out using the same questionnaires and study design [13]. The Italian PRISMA study reported a lower proportion of subjects with mental symptoms but it was carried out in younger subjects, used different tools, and collected data from questionnaires filled out by children’s parents [18]. The European multicenter study SEYLE reported the prevalence of emotional problems evaluated through SDQ in various countries, including Italy [19]. It showed a lower prevalence of emotional problems (11.8%) compared to the prevalence of internalizing symptoms scale found in our study (26.7%). A higher prevalence of depressive problems and unemployment among adults in Naples area may explain the higher prevalence of mental health symptoms among adolescents in Naples than Brescia in our study [12].

**Table 4:** Prevalence of depressive symptoms and symptoms of mental health problems among adolescents in the Be Teen and other surveys

	Be Teen	Kupol	SEYLE-Europe	PrISMA
Year of the survey	2018	2013–2015	2009–2010	2007
Area involved	Italy	Sweden	Italy	Italy
Number of participants	3001	3349	1192	3418
Age of participants	15–16	15–16	13–17	10–14
Questionnaire score indicative of mental symptoms/problems				
Total	SDQ: 17.8% CES-DC: 20.8%	SDQ: 18.5% CES-DC: 15.2%	SDQ: 11.8%^a^	CBCL: 9.8% DAWBA: 8.2%
Male	SDQ: 12.1% CES-DC:10.4%	SDQ: 12.9% CES-DC: 4.8%		
Female	SDQ: 22.3% CES-DC: 28.7%	SDQ: 23.6 CES-DC: 24.6%		

^a^Emotional problems. *PrISMA* Progetto Italiano Salute Mentale Adolescenti [Italian Project on Adolescents’ Mental Health]

Tobacco smoking and alcohol consumption prevalence rates in the present study (see Table 1) are not far from those observed in two large studies carried out among Italian children in 2015–2018. In fact, students who had smoked at least one cigarette in the past month were 40% and 35% in the ESPAD study [20], Italian participants (mean age 15.2 years, n = 4059), and 31.9% and 24.8% in the HBSC study [21] (mean age 15 years, n = 18918), in females and males, respectively. Students who had drunk at least one alcoholic beverage in the past month were 53% and 60% in the ESPAD study [20] for Italian participants, and 45.2% and 53.5% in the HBSC study [21], in females and males, respectively. Instead, a much lower prevalence of tobacco smoking and alcohol drinking was found in the Kupol study, probably depending on the restrictive national policy for substance use in Sweden, which is one of the countries with the lowest proportion of tobacco and alcohol users in adults in Europe at present [22].

In line with the literature, not only females showed a higher prevalence of depressive and internalizing symptoms than males [23, 24], but also had a higher prevalence of factors associated with symptoms of mental health problems as tobacco smoking, screen time, and physical and psychological bullying victimization.

Among factors associated with depression and other mental health problems, an association of tobacco smoking and alcohol consumption with mental health problems in adolescence has been observed in most studies [4, 5]. This association should be considered bidirectional: tobacco smoking and alcohol drinking may be facilitated by depressive symptoms as a coping mechanism [25, 26], but these substances have also a well-recognized biological effect on the maturating adolescent’s brain and may influence the subsequent development of depression [27, 28].

The school environment has a deep influence on students’ behavior and well-being and a relationship between students’ perception of the school context and their mental health has been recognized [6, 7]. Both disruptive environments characterized by physical and psychological bullying and competitive environments with high academic standards may impact students’ well-being and development leading to symptoms of mental health problems. In the present study, we found an association between students’ perception of unsatisfying school climate and symptoms of mental health problems, unlike the Kupol study that found the association only for teachers-perceived school climate [29]. Although similar associations have been observed between the other risk factors investigated and students’ mental health in the Kupol and Be-Teen study, the apparent discrepancy may be explained by differences in organization and educational methods. Future prospective studies should aim to unravel the underlying pattern that links school climate and mental health problems among adolescents.

The widespread use of technology tools during leisure time by adolescents, particularly social media, has arisen concern as regards the risk of mental health problems. Our findings are in agreement with some recent studies showing an association between digital technology use and depression [11, 30, 31]. However, other studies found only a weak association between digital technology use and adolescent well-being [32]. Despite plenty of epidemiologic studies, the overall evidence of an association between screen time and symptoms of mental health problems is still uncertain: among 7 systematic reviews (3 meta-analyses) that selected 11–70 studies and 5582–46,015 participants, only 2 reported significant associations between social media and depressive symptoms [33]. The type of screen time may play also a main role. In the present study, we found that time spent on TV and social media was more strongly associated with symptoms of mental health problems than web navigation and playing videogames, similarly to findings from other studies [10, 31]. Overall, these results suggest that the effect of screen time on adolescents’ mental well-being depends on the type and content: it is more explained by the “upward social comparison” (screen time users compare themselves with images of “perfect” individuals) and “reinforcing spirals” (screen time users seek information confirming their depressive status) hypotheses than by the “displacement hypothesis” (screen time displaces time participating in healthier activities [10].

Bullying is one of the most common expressions of violence by peers in the school context [34]. The prevalence rates of bullying vary widely across studies, according to its definition, time period, gender, age, social context, and other variables [34]. Prevalence rates of 35% for bullying perpetration and 36% for bullying victimization were estimated in a meta-analysis of 80 studies that reported similar prevalence rates for cyber and traditional bullying and/or aggression in adolescents [35]. Accordingly, we found that about 40% of students claimed to have been bullying authors or victims, more often psychological than physical bullying, in agreement with another Italian study [36]. We found a strong association between bullying and symptoms of mental health problems at both scales, more for bullying victimization than active bullying, in line with a national, population-based Australian study showing that adolescents involved in bullying had significantly increased SDQ depression and anxiety scores in all bullying roles and types [37]. Other studies have shown a concurrent association between involvement in bullying and depression in adolescents, although results are not consistent, as the relationship may be influenced by many factors and because depression by itself may predispose adolescents to victimization from bullying [8].

### Strengths and limitations

The main strength of our study is the assessment of the prevalence of depressive symptoms, internalizing and externalizing problems, and their associations with various adolescents’ common habits using standardized methods in a relatively large sample of students in two different areas of the country.

For assessing mental health problems, we used two internationally validated tools for screening of symptoms of mental health problems in adolescence, the SDQ and CES-DC. We administered anonymous questionnaires during school time to whole classes to minimize the risk of selection and information biases. Indeed, the high student’s response rate and a low proportion of questionnaires discharged support a low risk of selection and information bias. Internal validity was also confirmed by the high values of Cronbach’s alpha for both CES-DC and SDQ:

Some limitation should also be acknowledged. The cross-sectional design is the main limitation of our study as it did not allow us to evaluate the direction of the cause-effect relationships. However, prospective cohort studies which aim to assess the associations between some risk-taking behaviors such as substance use and mental health are at risk of selection bias and incorrect answers, as adolescents must provide informed consent for participating in the study and answer to questions of non-anonymous questionnaires. Indeed, in the Kupol study, a selection of participants has occurred with less than 30% response rate, which may have determined an estimate of the low prevalence of both substance use and depressive symptoms in participating students as compared to the Swedish general population.

Comparing Naples and Brescia areas, the prevalence of tobacco smoking was higher among adults in the former, according to a national survey [12] while it was lower among adolescents in this study. This might be due to possible higher underreporting of tobacco smoking by students in Naples than Brescia area in our study.

Finally, the risk of confounding in evaluating factors associated with mental health in adolescence is of concern, as the underlying causal pathway is complex and includes several factors. In this study, we investigated only some factors of the many possibilities related to mental health problems. Yet, we found that all the main factors assessed in our study were associated with students’ mental health problems also considering multivariable models, without substantial changes in the OR estimates. Furthermore, analyses stratified for gender and center yielded similar results. Finally, the prevalences observed in the present study were substantially similar to those of other surveys, supporting for external validity of this study.

## Conclusions

The present study shows a relatively high prevalence of symptoms of mental health problems, particularly in females, and their association with tobacco and alcohol use, screen time, bullying, and school climate in a large sample of Italian 15–16 years old adolescents. These findings highlight the opportunity for preventive interventions at school, since the investigated risk factors for depression and internalizing problems are theoretically modifiable [34]. The present COVID-19 pandemic has produced deep changes in adolescents’ habits, particularly social deprivation, with negative consequences on their mental health, as already observed [38]. Therefore, it could be of the highest interest to repeat cross-sectional studies on adolescents’ mental health, like this one, in the future to investigate the possible changes from before to after the COVID-19 pandemic time.

## Supplementary Information

**Additional file 1: Supplementary Table 1**. Socio-demographic characteristics, CES-DC and SDQ abnormal scores and risk factors for symptoms of mental health problems of all students, separated between sexes and areas (Brescia and Naples). **Supplementary Table 2**. Number and proportion of students with, and odds ratio for, depressive symptoms on CES-DC among females according to behavioral factors and school climate perception. **Supplementary Table 3**. Number and proportion of students with, and odds ratio for, depressive symptom on CES-DC among males according to behavioral factors and school climate perception. **Supplementary Table 4**. Number and proportion of students with, and odds ratio for, symptoms of mental health problems on SDQ among females according to behavioral factors and school climate perception. **Supplementary Table 5**. Number and proportion of students with, and odds ratio for, symptoms of mental health problems on SDQ among males according to behavioral factors and school climate perception.

## Data Availability

The dataset analyzed during the current study are not publicly available in agreement with the ethical recommendations but is available from the corresponding author on reasonable request.

## Abbreviations

CES-DC: Center for Epidemiological Studies–Depression Scale for Children
SDQ: Strengths and Difficulties Questionnaire
PESOC: Pedagogical and Social Climate of a school
OR: Odds ratios
CI: Confidence interval
SD: Standard deviation
