# Supplementary material for: Symptoms of mental health problems among Italian adolescents in 2017–2018 school year: a multicenter cross-sectional study
Source: Environ Health Prev Med. 2021 Jun 21;26:67. doi: 10.1186/s12199-021-00988-4 (PMC8216089; doi:10.1186/s12199-021-00988-4)
Supplement: Supplementary file 1 — Additional file 1: Supplementary Table 1. Socio-demographic characteristics, CES-DC and SDQ abnormal scores and risk factors for symptoms of mental health problems of all students, separated between sexes and areas (Brescia and Naples). Supplementary Table 2. Number and proportion of students with, and odds ratio for, depressive symptoms on CES-DC among females according to behavioral factors and school climate perception. Supplementary Table 3. Number and proportion of students with, and odds ratio for, depressive symptom on CES-DC among males according to behavioral factors and school climate perception. Supplementary Table 4. Number and proportion of students with, and odds ratio for, symptoms of mental health problems on SDQ among females according to behavioral factors and school climate perception. Supplementary Table 5. Number and proportion of students with, and odds ratio for, symptoms of mental health problems on SDQ among males according to behavioral factors and school climate perception. [file 12199_2021_988_MOESM1_ESM.docx]

**Supplementary Table 1. Socio-demographic characteristics, CES-DC and SDQ abnormal scores and risk factors for symptoms of mental health problems of all students, separated between sexes and areas (Brescia and Naples)**

|  | **Females** | |  | **Males** | |  |
| --- | --- | --- | --- | --- | --- | --- |
|  | **Brescia** | **Napoli** |  | **Brescia** | **Napoli** |  |
|  | **n=1056** | **n=623** | ***p* value** | **n=906** | **n=417** | ***p* value** |
|  | **n (%)** | **n (%)** |  | **n (%)** | **n (%)** |  |
| **Socio-demographic characteristics** |  |  |  |  |  |  |
| Mean age (SD), years | 15.2 (0.4) | 15.2 (0.4) | 0.918 | 15.2 (0.4) | 15.2 (0.4) | 0.443 |
| Parental highest school, years |  |  |  |  |  |  |
| ≤8 | 145 (14.1) | 104 (17.5) |  | 110 (12.8) | 70 (17.8) |  |
| 9 - 13 | 526 (51.1) | 267 (44.9) |  | 406 (47.1) | 140 (35.6) |  |
| ≥14 | 359 (34.8) | 223 (37.6) | 0.065 | 345 (40.1) | 183 (46.6) | 0.018 |
|  |  |  |  |  |  |  |
| **Mental health evaluation** |  |  |  |  |  |  |
| CES-DC scale |  |  |  |  |  |  |
| Mean score (SD) | 21.6 (12.6) | 24.0 (12.9) | 0.005 | 13.9 (10.6) | 17.1 (11.3) | <0.001 |
| Depressive symptoms | 253 (26.5) | 179 (32.4) | 0.015 | 70 (8.8) | 49 (14.1) | 0.007 |
| SDQ scale |  |  |  |  |  |  |
| Mean score (SD) | 14.3 (6.0) | 15.2 (6.2) | 0.009 | 11.5 (5.7) | 13.1 (6.5) | 0.002 |
| Symptoms of mental health problems | 213 (20.5) | 153 (25.3) | 0.047 | 83 (9.3) | 72 (18.5) | <0.001 |
| SDQ, internalizing subscale |  |  |  |  |  |  |
| Mean score (SD) | 7.1 (3.8) | 7.5 (3.8) | 0.064 | 4.6 (3.5) | 5.7 (3.8) | <0.001 |
| Internalizing symtpoms | 355 (33.9) | 225 (37.0) | 0.203 | 124 (13.9) | 83 (21.1) | <0.001 |
| SDQ, externalizing subscale |  |  |  |  |  |  |
| Mean score (SD) | 7.2 (3.5) | 7.7 (3.6) | 0.027 | 6.9 (3.5) | 7.44 (3.7) | 0.026 |
| Externalizing symptoms | 183 (17.5) | 143 (23.3) | 0.004 | 132 (14.7) | 79 (20.0) | 0.018 |
|  |  |  |  |  |  |  |
| **Substance use** |  |  |  |  |  |  |
| Tobacco smoking | 349 (33.1) | 180 (28.9) | 0.080 | 283 (31.2) | 100 (24.2) | 0.009 |
| Perceived tobacco dependence | 156 (15.1) | 83 (13.9) | 0.528 | 103 (11.5) | 37 (9.2) | 0.219 |
| Alcohol consumption | 407 (39.1) | 179 (29.7) | <0.001 | 434 (48.4) | 169 (42.0) | 0.033 |
| Perceived drunkenness | 75 (7.3) | 23 (3.9) | 0.005 | 84 (9.5) | 23 (5.8) | 0.024 |
|  |  |  |  |  |  |  |
| **Screen time** |  |  |  |  |  |  |
| TV |  |  |  |  |  |  |
| 0-1 h | 549 (53.1) | 286 (47.5) |  | 561 (63.6) | 225 (56.5) |  |
| 2-3 h | 289 (27.9) | 159 (26.4) |  | 213 (24.2) | 89 (22.4) |  |
| ≥4 h | 196 (19.0) | 157 (26.1) | <0.001 | 108 (12.2) | 84 (21.1) | <0.001 |
| Social networks |  |  |  |  |  |  |
| 0-1 h | 253 (24.4) | 71 (11.7) |  | 14.39 (49.2) | 131 (32.8) |  |
| 2-3 h | 301 (28.9) | 92 (15.2) |  | 255 (28.5) | 73 (18.2) |  |
| 4-5 h | 258 (24.8) | 161 (26.5) |  | 134 (15.0) | 101 (25.2) |  |
| ≥6 h | 228 (21.9) | 283 (46.6) | <0.001 | 65 (7.3) | 95 (23.8) | <0.001 |
| Web navigation |  |  |  |  |  |  |
| 0-1 h | 814 (79.3) | 397 (65.6) |  | 748 (84.9) | 302 (76.8) |  |
| 2-3 h | 142 (13.8) | 116 (19.2) |  | 99 (11.2) | 50 (12.7) |  |
| ≥4 h | 71 (6.9) | 92 (15.2) | <0.001 | 34 (3.9) | 41 (10.4) | <0.001 |
| Videogames |  |  |  |  |  |  |
| 0-1 h | 977 (94.9) | 554 (91.6) |  | 544 (61.5) | 179 (44.6) |  |
| 2-3 h | 27 (2.6) | 23 (3.8) |  | 193 (21.8) | 99 (24.7) |  |
| ≥4 h | 25 (2.5) | 28 (4.6) | 0.015 | 148 (16.7) | 123 (30.7) | <0.001 |
|  |  |  |  |  |  |  |
| **Bullying** |  |  |  |  |  |  |
| Bullying |  |  |  |  |  |  |
| Victims | 471 (44.8) | 257 (41.8) | 0.236 | 342 (38.0) | 160 (39.4) | 0.618 |
| Authors | 380 (36.2) | 171 (27.7) | <0.001 | 446 (49.9) | 182 (45.1) | 0.102 |
| Physical |  |  |  |  |  |  |
| Victims | 53 (5.1) | 25 (4.1) | 0.359 | 80 (8.9) | 40 (10.0) | 0.553 |
| Authors | 62 (5.9) | 37 (6.0) | 0.932 | 165 (18.6) | 75 (19.1) | 0.846 |
| Psychological |  |  |  |  |  |  |
| Victims | 410 (39.4) | 227 (37.2) | 0.382 | 261 (29.1) | 117 (29.1) | 0.998 |
| Authors | 314 (30.1) | 131 (21.4) | <0.001 | 276 (31.2) | 103 (26.2) | 0.074 |
|  |  |  |  |  |  |  |
| **School climate** |  |  |  |  |  |  |
| PESOC scale | 359 (34.0) | 208 (33.4) | 0.799 | 308 (34.0) | 107 (25.8) | 0.003 |

P-Value refers to ꭓ^2^ test, test for linear trends of the log-odds. Percentage of students with negative perceptions of school climate

**Supplementary Table 2. Number and proportion of students with, and odds ratio for, depressive symptoms on CES-DC among females according to behavioral factors and school climate perception.**

| **Females** |  | **n (%)** | **Unadjusted models** | **Adjusted models A** | **Adjusted models B** |
| --- | --- | --- | --- | --- | --- |
|  |  |  | **OR (95% CI)** | **OR (95% CI)** | **OR (95% CI)** |
| **Substance use** |  |  |  |  |  |
| Tobacco smoking | No | 236 (23.2) | 1 | 1 | 1 |
|  | Yes | 196 (40.1) | 2.2 (1.8,2.8) | 2.0 (1.6,2.6) | 2.1 (1.6,2.7) |
| Perceived tobacco dependence | No | 310 (24.9) | 1 | 1 | 1 |
|  | Yes | 101 (44.9) | 2.5 (1.8,3.3) | 2.2 (1.6,3.0) | 2.2 (1.6,3.0) |
| Alcohol consumption | No | 244 (25.8) | 1 | 1 | 1 |
|  | Yes | 183 (33.9) | 1.5 (1.2,1.9) | 1.4 (1.1,1.8) | 1.4 (1.1,1.8) |
| Perceived drunkenness | No | 382 (27.8) | 1 | 1 | 1 |
|  | Yes | 40 (43.5) | 2.0 (1.3,3.1) | 2.0 (1.3,3.2) | 2.0 (1.3,3.2) |
|  |  |  |  |  |  |
| **Screen time** |  |  |  |  |  |
| TV |  |  |  |  |  |
| 0-3 h |  | 299 (25.8) | 1 | 1 | 1 |
| ≥4 h |  | 125 (38.8) | 1.8 (1.4,2.4) | 1.8 (1.4,2.4) | 1.8 (1.4,2.4) |
| Social networks |  |  |  |  |  |
| 0-3 h |  | 143 (22.4) | 1 | 1 | 1 |
| 4-5 h |  | 117 (29.9) | 1.5 (1.1,2.0) | 1.3 (1.0,1.8) | 1.3 (1.0,1.8) |
| ≥6 h |  | 163 (35.9) | 1.9 (1.5,2.5) | 1.7 (1.3,2.3) | 1.6 (1.2,2.2) |
| Web navigation |  |  |  |  |  |
| 0-3 h |  | 372 (27.9) | 1 | 1 | 1 |
| ≥4 h |  | 52 (36.4) | 1.5 (1.0,2.1) | 1.4 (1.0,2.1) | 1.4 (0.9,2.0) |
| Videogames |  |  |  |  |  |
| 0-3 h |  | 404 (28.3) | 1 | 1 | 1 |
| ≥4 h |  | 19 (38.0) | 1.6 (0.9,2.8) | 1.5 (0.8,2.7) | 1.4 (0.7,2.6) |
|  |  |  |  |  |  |
| **Bullying** |  |  |  |  |  |
| Bullying |  |  |  |  |  |
| Victims | No | 165 (19.8) | 1 | 1 | 1 |
|  | Yes | 265 (39.4) | 2.6 (2.1,3.3) | 2.6 (2.0,3.3) | 2.6 (2.1,3.4) |
| Author | No | 253 (25.5) | 1 | 1 | 1 |
|  | Yes | 177 (34.7) | 1.6 (1.2,2.0) | 1.3 (1.0,1.6) | 1.3 (1.0,1.6) |
| Physical bullying |  |  |  |  |  |
| Victims | No | 385 (27.1) | 1 | 1 | 1 |
|  | Yes | 39 (54.2) | 3.2 (2.0,5.1) | 3.2 (1.9,5.3) | 3.2 (1.9,5.4) |
| Author | No | 394 (28.1) | 1 | 1 | 1 |
|  | Yes | 32 (34.4) | 1.3 (0.9,2.1) | 1.0 (0.6,1.6) | 1.0 (0.6,1.6) |
| Psychological bullying |  |  |  |  |  |
| Victims | No | 204 (22.6) | 1 | 1 | 1 |
|  | Yes | 220 (37.3) | 2.0 (1.6,2.6) | 2.0 (1.6,2.6) | 2.0 (1.6,2.6) |
| Author | No | 285 (26.3) | 1 | 1 | 1 |
|  | Yes | 141 (34.3) | 1.5 (1.1,1.9) | 1.2 (0.9,1.6) | 1.2 (0.9,1.6) |
| **School climate** |  |  |  |  |  |
| Positive perception |  | 229 (22.9) | 1 | 1 | 1 |
| Negative perception |  | 203 (39.9) | 2.2 (1.8,2.8) | 1.9 (1.5,2.5) | 1.9 (1.5,2.5) |
|  |  |  |  |  |  |

Models A: adjusted for tobacco smoking social networks use, bullying victimization, parental education and sex. Models B: multilevel regression models for institutes and centers adjusted as Models A.

**Supplementary Table 3. Number and proportion of students with, and odds ratio for, depressive symptom on CES-DC among males according to behavioral factors and school climate perception.**

| **Males** |  | **n (%)** | **Unadjusted model** | **Adjusted models A** | **Adjusted models B** |  |
| --- | --- | --- | --- | --- | --- | --- |
|  |  |  | **OR (95% CI)** | **OR (95% CI)** | **OR (95% CI)** |  |
| **Substance use** |  |  |  |  |  |  |
| Tobacco smoking | No | 75 (9.3) | 1 | 1 | 1 |  |
|  | Yes | 44 (13.1) | 1.5 (1.0,2.2) | 1.4 (0.9,2.1) | 1.4 (0.9,2.1) |  |
| Perceived tobacco dependence | No | 89 (8.9) | 1 | 1 | 1 |  |
|  | Yes | 25 (20.3) | 2.6 (1.6,4.3) | 2.4 (1.4,4.1) | 2.4 (1.4,4.1) |  |
| Alcohol consumption | No | 55 (9.2) | 1 | 1 | 1 |  |
|  | Yes | 61 (11.4) | 1.3 (0.9,1.8) | 1.4 (0.9,2.1) | 1.4 (0,9,2.1) |  |
| Perceived drunkenness | No | 95 (9.4) | 1 | 1 | 1 |  |
|  | Yes | 16 (16.0) | 1.8 (1.0,3.3) | 1.7 (0.9,3.2) | 1.7 (0.9,3.2) |  |
|  |  |  |  |  |  |  |
| **Screen time** |  |  |  |  |  |  |
| TV |  |  |  |  |  |  |
| 0-3 h |  | 83 (8.6) | 1 | 1 | 1 |  |
| ≥4 h |  | 32 (20.1) | 2.7 (1.7,4.2) | 2.3 (1.4,3.8) | 2.3 (1.4,3.7) |  |
| Social networks |  |  |  |  |  |  |
| 0-3 h |  | 64 (8.0) | 1 | 1 | 1 |  |
| 4-5 h |  | 28 (14.0) | 1.9 (1.2,3.0) | 1.8 (1.1,3.0) | 1.8 (1.1,3.0) |  |
| ≥6 h |  | 25 (18.4) | 2.6 (1.6,4.3) | 2.4 (1.4,4.2) | 2.4 (1.4,4.2) |  |
| Web navigation |  |  |  |  |  |  |
| 0-3 h |  | 100 (9.5) | 1 | 1 | 1 |  |
| ≥4 h |  | 15 (23.4) | 2.9 (1.6,5.4) | 2.3 (1.2,4.5) | 2.2 (1.1,4.3) |  |
| Videogames |  |  |  |  |  |  |
| 0-3 h |  | 86 (9.6) | 1 | 1 | 1 |  |
| ≥4 h |  | 29 (12.5) | 1.3 (0.9,2.1) | 1.2 (0.7,1.9) | 1.1 (0.7,1.9) |  |
|  |  |  |  |  |  |  |
| **Bullying** |  |  |  |  |  |  |
| Bullying |  |  |  |  |  |  |
| Victims | No | 42 (6.0) | 1 | 1 | 1 |  |
|  | Yes | 75 (17.1) | 3.2 (2.2,4.8) | 3.1 (2.0,4.7) | 3.1 (2.0,4.7) |  |
| Author | No | 52 (8.8) | 1 | 1 | 1 |  |
|  | Yes | 63 (11.5) | 1.3 (0.9,2.0) | 1.2 (0.8,1.8) | 1.2 (0.8,1.8) |  |
| Physical bullying |  |  |  |  |  |  |
| Victims | No | 91 (8.8) | 1 | 1 | 1 |  |
|  | Yes | 25 (24.8) | 3.4 (2.1,5.6) | 3.4 (2.0,5.8) | 3.4 (2.0,5.9) |  |
| Author | No | 77 (8.4) | 1 | 1 | 1 |  |
|  | Yes | 31 (15.4) | 2.0 (1.3,3.1) | 1.9 (1.2,3.1) | 1.9 (1.2,3.1) |  |
| Psychological bullying |  |  |  |  |  |  |
| Victims | No | 67 (8.4) | 1 | 1 | 1 |  |
|  | Yes | 49 (14.5) | 1.9 (1.3,2.8) | 1.8 (1.2,2.8) | 1.8 (1.2,2.8) |  |
| Author | No | 80 (10.2) | 1 | 1 | 1 |  |
|  | Yes | 28 (8.3) | 0.8 (0.5,1.2) | 0.7 (0.4,1.1) | 0.7 (0.4,1.1) |  |
|  |  |  |  |  |  |  |
| **School climate** |  |  |  |  |  |  |
| Positive perception |  | 64 (8.1) | 1 | 1 | 1 |  |
| Negative perception |  | 55 (15.3) | 2.0 (1.4,3.0) | 2.0 (1.3,3.0) | 2.0 (1.3,3.1) |  |

Models A: adjusted for tobacco smoking, social networks use, bullying victimization, parental education and sex. Models B: multilevel regression models for institutes and centers adjusted as Models A.

**Supplementary Table 4. Number and proportion of students with, and odds ratio for, symptoms of mental health problems on SDQ among females according to behavioral factors and school climate perception.**

| **Females** |  | **N (%)** | **Unadjusted models** | **Adjusted models A** | **Adjusted models B** |
| --- | --- | --- | --- | --- | --- |
|  |  |  | **OR (95% CI)** | **OR (95% CI)** | **OR (95% CI)** |
| **Substance use** |  |  |  |  |  |
| Tobacco smoking | No | 210 (18.7) | 1 | 1 | 1 |
|  | Yes | 156 (29.9) | 1.9 (1.5,2.4) | 1.7 (1.3,2.2) | 1.7 (1.3,2.2) |
| Perceived tobacco dependence | No | 265 (19.4) | 1 | 1 | 1 |
|  | Yes | 88 (37.6) | 2.5 (1.9,3.4) | 2.3 (1.6,3.1) | 2.3 (1.6,3.1) |
| Alcohol consumption | No | 202 (19.5) | 1 | 1 | 1 |
|  | Yes | 155 (26.8) | 1.5 (1.2,1.9) | 1.4 (1.1,1.8) | 1.4 (1.1,1.8) |
| Perceived drunkenness | No | 320 (21.4) | 1 | 1 | 1 |
|  | Yes | 31 (31.6) | 1.7 (1.1,2.7) | 1.7 (1.1,2.8) | 1.7 (1.1,2.8) |
|  |  |  |  |  |  |
| **Screen time** |  |  |  |  |  |
| TV |  |  |  |  |  |
| 0-3 h |  | 245 (19.4) | 1 | 1 | 1 |
| ≥4 h |  | 115 (33.2) | 2.1 (1.6,2.7) | 2.2 (1.7,3.0) | 2.2 (1.7,2.9) |
| Social networks |  |  |  |  |  |
| 0-3 h |  | 119 (17.0) | 1 | 1 | 1 |
| 4-5 h |  | 83 (20.1) | 1.2 (0.9,1.7) | 1.1 (0.8,1.6) | 1.1 (0.8,1.6) |
| ≥6 h |  | 157 (31.1) | 2.2 (1.7,2.9) | 2.0 (1.5,2.7) | 2.0 (1.5,2.7) |
| Web navigation |  |  |  |  |  |
| 0-3 h |  | 308 (21.3) | 1 | 1 | 1 |
| ≥4 h |  | 49 (30.8) | 1.7 (1.2,2.4) | 1.6 (1.1,2.3) | 1.5 (1.0,2.2) |
| Videogames |  |  |  |  |  |
| 0-3 h |  | 334 (21.5) | 1 | 1 | 1 |
| ≥4 h |  | 20 (39.2) | 2.4 (1.3,4.2) | 2.1 (1.1,3.9) | 2.0 (1.1,3.8) |
|  |  |  |  |  |  |
| **Bullying** |  |  |  |  |  |
| Bullying |  |  |  |  |  |
| Victims | No | 132 (14.3) | 1 | 1 | 1 |
|  | Yes | 230 (32.2) | 2.9 (2.2,3.6) | 2.9 (2.3,3.8) | 2.9 (2.3,3.8) |
| Author | No | 185 (16.9) | 1 | 1 | 1 |
|  | Yes | 177 (32.7) | 2.4 (1.9,3.0) | 2.1 (1.6,2.7) | 2.1 (1.6,2.7) |
| Physical bullying |  |  |  |  |  |
| Victims | No | 309 (20.0) | 1 | 1 | 1 |
|  | Yes | 48 (61.5) | 6.4 (4.0,10.3) | 5.5 (3.3,9.0) | 5.5 (3.3,9.0) |
| Author | No | 317 (20.7) | 1 | 1 | 1 |
|  | Yes | 45 (46.9) | 3.4 (2.2,5.2) | 2.7 (1.7,4.3) | 2.7 (1.7,4.3) |
| Psychological bullying |  |  |  |  |  |
| Victims | No | 180 (18.0) | 1 | 1 | 1 |
|  | Yes | 177 (28.3) | 1.8 (1.4,2.3) | 1.9 (1.5,2.5) | 1.9 (1.5,2.5) |
| Author | No | 230 (19.3) | 1 | 1 | 1 |
|  | Yes | 132 (30.1) | 1.8 (1.4,2.3) | 1.6 (1.2,2.1) | 1.6 (1.2,2.1) |
| **School climate** |  |  |  |  |  |
| Positive perception |  | 194 (17.8) | 1 | 1 | 1 |
| Negative perception |  | 172 (31.1) | 2.1 (1.6,2.6) | 1.8 (1.4,2.4) | 1.8 (1.4,2.4) |

Models A: adjusted for tobacco smoking, social networks use, bullying victimization, parental education and sex. Models B: multilevel regression models for institutes and centers adjusted as Models A.

**Supplementary Table 5. Number and proportion of students with, and odds ratio for, symptoms of mental health problems on SDQ among males according to behavioral factors and school climate perception.**

| **Males** |  | **N (%)** | **Unadjusted models** | **Adjusted models A** | **Adjusted models B** |
| --- | --- | --- | --- | --- | --- |
|  |  |  | **OR (95% CI)** | **OR (95% CI)** | **OR (95% CI)** |
| **Substance use** |  |  |  |  |  |
| Tobacco smoking | No | 91 (10.0) | 1 | 1 | 1 |
|  | Yes | 64 (17.2) | 1.9 (1.3,2.6) | 1.7 (1.1,2.4) | 1.8 (1.2,2.6) |
| Perceived tobacco dependence | No | 117 (10.4) | 1 | 1 | 1 |
|  | Yes | 32 (23.2) | 2.6 (1.7,4.0) | 2.8 (1.7,4.5) | 2.8 (1.8,4.6) |
| Alcohol consumption | No | 75 (11.1) | 1 | 1 | 1 |
|  | Yes | 77 (13.1) | 1.2 (0.9,1.7) | 1.2 (0.8,1.8) | 1.2 (0.8,1.8) |
| Perceived drunkenness | No | 131 (11.5) | 1 | 1 | 1 |
|  | Yes | 17 (16.4) | 1.5 (0.9,2.6) | 1.1 (0.6,2.1) | 1.1 (0.6,2.1) |
|  |  |  |  |  |  |
| **Screen time** |  |  |  |  |  |
| TV |  |  |  |  |  |
| 0-3 h |  | 104 (9.8) | 1 | 1 | 1 |
| ≥4 h |  | 46 (24.6) | 3.0 (2.0,4.4) | 2.9 (1.9,4.5) | 2.7 (1.7,4.2) |
| Social networks |  |  |  |  |  |
| 0-3 h |  | 80 (9.1) | 1 | 1 | 1 |
| 4-5 h |  | 40 (17.6) | 2.1 (1.4,3.2) | 2.2 (1.4,3.4) | 2.0 (1.3,3.1) |
| ≥6 h |  | 34 (22.4) | 2.9 (1.9,4.5) | 2.6 (1.6,4.3) | 2.2 (1.3,3.8) |
| Web navigation |  |  |  |  |  |
| 0-3 h |  | 128 (10.9) | 1 | 1 | 1 |
| ≥4 h |  | 21 (29.2) | 3.4 (2.0,5.8) | 3.5 (1.9,6.3) | 2.9 (1.6,5.4) |
| Videogames |  |  |  |  |  |
| 0-3 h |  | 105 (10.6) | 1 | 1 | 1 |
| ≥4 h |  | 46 (17.6) | 1.8 (1.2,2.6) | 1.7 (1.1,2.6) | 1.5 (1.0,2.4) |
|  |  |  |  |  |  |
| **Bullying** |  |  |  |  |  |
| Bullying |  |  |  |  |  |
| Victims | No | 54 (6.9) | 1 | 1 | 1 |
|  | Yes | 98 (20.0) | 3.4 (2.4,4.8) | 3.6 (2.4,5.3) | 3.7 (2.5,5.5) |
| Author | No | 51 (7.8) | 1 | 1 | 1 |
|  | Yes | 100 (16.3) | 2.3 (1.6,3.3) | 2.1 (1.4,3.0) | 2.1 (1.4,3.1) |
| Physical bullying |  |  |  |  |  |
| Victims | No | 111 (9.6) | 1 | 1 | 1 |
|  | Yes | 40 (34.5) | 4.9 (3.2,7.6) | 4.7 (2.9,7.6) | 4.7 (2.9,7.6) |
| Author | No | 92 (9.0) | 1 | 1 | 1 |
|  | Yes | 51 (22.0) | 2.8 (1.9,4.1) | 2.7 (1.8,4.1) | 2.7 (1.8,4.2) |
| Psychological bullying |  |  |  |  |  |
| Victims | No | 94 (10.5) | 1 | 1 | 1 |
|  | Yes | 57 (15.4) | 1.6 (1.1,2.2) | 1.7 (1.2,2.6) | 1.8 (1.2,2.6) |
| Author | No | 98 (11.2) | 1 | 1 | 1 |
|  | Yes | 45 (12.1) | 1.1 (0.8,1.6) | 1.0 (0.7,1.5) | 1.1 (0.7,1.6) |
| **School climate** |  |  |  |  |  |
| Positive perception |  | 89 (10.2) | 1 | 1 | 1 |
| Negative perception |  | 66 (16.2) | 1.7 (1.2,2.4) | 1.5 (1.0,2.2) | 1.7 (1.1,2.5) |

Models A: adjusted for tobacco smoking, social networks use, bullying victimization, parental education and sex. Models B: multilevel regression models for institutes and centers adjusted as Models A.
